# Integrated Metabolome, Transcriptome and Long Non-Coding RNA Analysis Reveals Potential Molecular Mechanisms of Sweet Cherry Fruit Ripening

**DOI:** 10.3390/ijms25189860

**Published:** 2024-09-12

**Authors:** Gangshuai Liu, Daqi Fu, Xuwei Duan, Jiahua Zhou, Hong Chang, Ranran Xu, Baogang Wang, Yunxiang Wang

**Affiliations:** 1 Institute of Agri-Food Processing and Nutrition, Beijing Academy of Agriculture and Forestry Sciences, Beijing 100097, China; lgsliugangshuai@cau.edu.cn (G.L.); zhoujiahua@yeah.net (J.Z.); changhongch2008@163.com (H.C.); xuranran@iapn.org.cn (R.X.); 2 College of Food Science & Nutritional Engineering, China Agricultural University, Beijing 100083, China; daqifu@cau.edu.cn; 3 Institute of Forestry and Pomology Sciences, Beijing Academy of Agriculture and Forestry Sciences, Beijing 100093, China; dxwlly@163.com

**Keywords:** *Prunus avium* L., abscisic acid, cell wall, anthocyanin, transcription factor

## Abstract

Long non-coding RNAs (lncRNAs), a class of important regulatory factors for many biological processes in plants, have received much attention in recent years. To explore the molecular roles of lncRNAs in sweet cherry fruit ripening, we conducted widely targeted metabolome, transcriptome and lncRNA analyses of sweet cherry fruit at three ripening stages (yellow stage, pink stage, and dark red stage). The results show that the ripening of sweet cherry fruit involves substantial metabolic changes, and the rapid accumulation of anthocyanins (cyanidin 3-rutinoside, cyanidin 3-O-galactoside, and cyanidin 3-O-glucoside) is the main cause of fruit coloration. These ripening-related alterations in the metabolic profile are driven by specific enzyme genes related to the synthesis and decomposition of abscisic acid (ABA), cell wall disintegration, and anthocyanin biosynthesis, as well as transcription factor genes, such as *MYBs*, *bHLHs*, and *WD40s*. LncRNAs can target these ripening-related genes to form regulatory modules, incorporated into the sweet cherry fruit ripening regulatory network. Our study reveals that the lncRNA-mRNA module is an important component of the sweet cherry fruit ripening regulatory network. During sweet cherry fruit ripening, the differential expression of lncRNAs will meditate the spatio-temporal specific expression of ripening-related target genes (encoding enzymes and transcription factors related to ABA metabolism, cell wall metabolism and anthocyanin metabolism), thus driving fruit ripening.

## 1. Introduction

Sweet cherry (*Prunus avium* L.) is one of the most favored fruit around the world due to its wonderful taste, attractive color and abundant nutrients, such as dietary fiber, phenolic acids, vitamin C, and anthocyanins [1]. As a non-climacteric fruit, sweet cherry is a potentially valuable model for studying fruit ripening due to its typical ripening characteristics, such as coloration, texture softening, and flavor compound accumulation [2,3], which involves extensive metabolic changes, and is driven by a series of complex molecular events, including spatio-temporal specific expression of ripening-related structural genes, plant hormone interactions (abscisic acid (ABA) as the core phytohormone), regulation at transcriptional and post-transcriptional levels, and epigenetic modification [2,4,5].

Aside from coding genes, non-coding RNAs (ncRNAs), comprising a considerable fraction of the plant genome, also perform essential regulatory functions in multiple biological processes [6]. Long ncRNAs (lncRNAs), a category of ncRNAs, are usually composed of more than 200 nucleotides (nt). Based on the characteristics of genomic position, lncRNAs are further categorized into four types: (i) intergenic lncRNAs (lincRNAs), (ii) antisense lncRNAs, (iii) intronic lncRNAs, and (iiii) sense lncRNAs [7]. For plants, lncRNAs are mostly transcribed via RNA polymerase II (Pol II), with a small amount transcribed by Pol III/IV/V [8]. lncRNAs could control gene transcription level in a *cis*- or *trans*-way, involving chromatin remodeling, transcriptional regulation, and post-transcriptional regulation (acting as precursors of microRNAs (miRNAs), interfering with alternative splicing, and competing with miRNAs for target genes) [9].

With ongoing advancement of sequencing technologies and bioinformatics, an increasing amount of lncRNAs were successively identified in different fruit species, including tomato [10], kiwifruit [11], sea-buckthorn [12], bell pepper [13], strawberry [14], banana [15], apple [16], melon [17], grapevine [18], pitaya [19], orange [20], avocado [21], lemon [22], cucumber [23], highbush blueberry [24], pomegranate [25], peach [26], black pepper [27], *Diospyros oleifera* [28], lychee [29], and pear [30].

Growing evidence indicates that lncRNAs play an essential part in modulating fruit ripening. For instance, tomato *lncRNA2155* can serve as a downstream direct target of the transcription factor ripening inhibitor (RIN), and knockout of *lncRNA2155* resulted in decreased expression of ethylene and carotenoid production-related genes, thus inhibiting fruit ripening [31]. In sea buckthorn, LNC1 and LNC2 can participate in the target competition of the anthocyanin biosynthesis regulatory modules miR156a-*SPL9* and miR828a-*MYB114*, to inhibit and promote the anthocyanin buildup throughout fruit ripening, respectively [12]. In apple, *MdLNC499*-mediated MdWRKY1-MdLNC499-MdERF109 transcriptional cascade promotes light-induced fruit coloration from the early stage to full ripeness [32]. FRUIT RIPENING RELATED LONG INTERGENIC RNA (FRILAIR) in strawberry can compete with miR397 for the target *LAC11a*, which encodes a laccase protein, promoting the transcription level of *LAC11a* and anthocyanin production as the fruit ripens [33].

However, the involvement of lncRNAs in the ripening process of sweet cherry fruit has yet to be adequately investigated. In this study, we conducted widely targeted metabolome detection in sweet cherry fruit for three ripening stages (yellow, pink, and dark red stage) to analyze the metabolic changes during fruit ripening, especially for flavonoid metabolism, and we profiled the expression patterns of mRNAs and lncRNAs of sweet cherry fruit at these three ripening stages by using transcriptome and lncRNA sequencing. In addition, we performed target gene prediction for lncRNAs. By integrating the results of the above analyses, the potential molecular mechanisms of lncRNA-mediated sweet cherry fruit ripening were revealed.

## 2. Results

### 2.1. Widely Targeted Metabolomics Analysis

**Figure 1:**
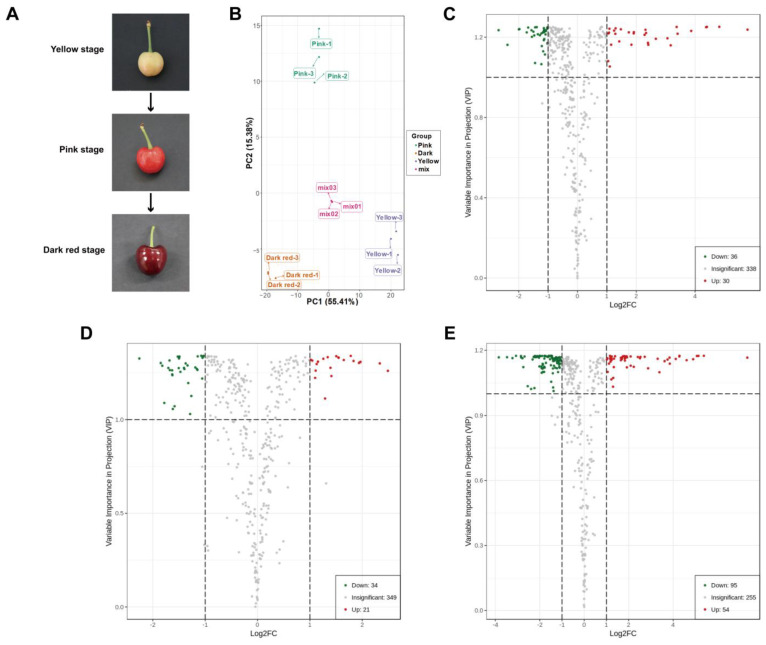
Analysis of differential metabolites in sweet cherry fruit at yellow stage, pink stage, and dark red stage. (**A**) The appearance of sweet cherry fruit at different ripening stages. (**B**) PCA of metabolites in sweet cherry fruit at different ripening stages. (**C**) Volcano plot of differential metabolites in sweet cherry fruit at yellow stage and pink stage. (**D**) Volcano plot of differential metabolites in sweet cherry fruit at pink stage and dark red stage. (**E**) Volcano plot of differential metabolites in sweet cherry fruit at yellow stage and dark red stage.

An overall of 404 metabolites were identified by widely targeted metabolomics in the three groups of sweet cherry fruit at different ripening periods (yellow stage, pink stage, and dark red stage), mainly including alkaloids, flavonoids, lignans and coumarins, lipid, terpene, tannins, organic acids, phenolic acids, amino acid and its derivatives, nucleotide and its derivates (Figure 1A; Table S1). PCA displayed good intra-group repeatability and significant inter-group variability among the three groups of sweet cherry fruit samples (Figure 1B). Compared with fruit at the yellow stage, there were 66 differential metabolites in fruit at the pink stage, of which 36 metabolites were down-regulated, and 30 metabolites were up-regulated (Figure 1C; Table S2). In comparison to pink stage fruit, there were 55 differential metabolites in dark red stage fruit, including 34 down-regulated metabolites and 21 up-regulated metabolites (Figure 1D; Table S3). When dark red stage fruit were compared with yellow stage fruit, 149 differential metabolites were observed, among which 95 metabolites were down-regulated, and 54 metabolites were up-regulated (Figure 1E; Table S4). Among these detected metabolites, alkaloids, organic acids, and tannins were closely associated with flavor changes through sweet cherry fruit ripening, and all showed a decreasing trend in overall abundance during fruit ripening (Table S1). The anthocyanins in flavonoids are the main pigments accumulated during sweet cherry fruit ripening, mainly including cyanidin 3-rutinoside, cyanidin 3-O-galactoside, cyanidin 3-O-glucoside, peonidin 3-O-glucoside chloride, cyanidin O-syringic acid, and cyanin chloride, among which cyanidin 3-rutinoside, cyanidin 3-O-galactoside, and cyanidin 3-O-glucoside were the most abundant, and the abundance of these anthocyanins all tended to increase during fruit ripening (Table S1).

**Figure 2:**
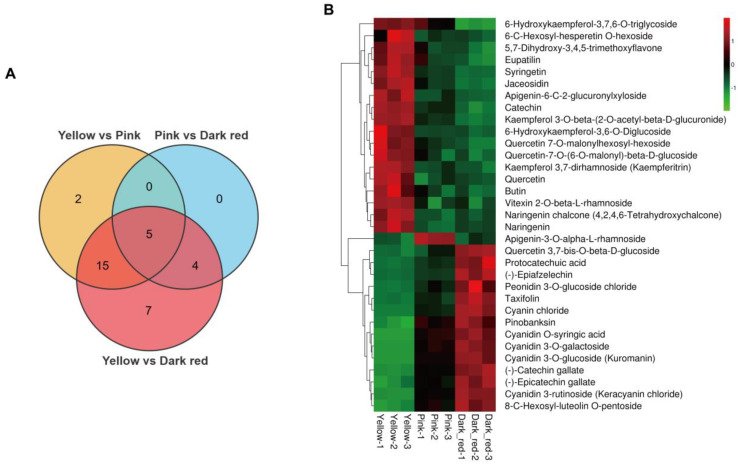
Analysis of differential flavonoids in sweet cherry fruit at yellow stage, pink stage, and dark red stage. (**A**) Venn diagram of differential flavonoids in sweet cherry fruit at different ripening stages. (**B**) Abundance heatmap of differential flavonoids in sweet cherry fruit at different ripening stages.

For flavonoid metabolism, a sum of 76 flavonoids were identified (Table S1). Compared to the yellow stage fruit, 22 differential flavonoids appeared in the fruit at the pink stage, of which 10 flavonoids were up-regulated (containing the six major anthocyanins: cyanidin 3-rutinoside, cyanidin 3-O-galactoside, cyanidin 3-O-glucoside, peonidin 3-O-glucoside chloride, cyanidin O-syringic acid, and cyanin chloride), and 12 flavonoids were down-regulated (containing two anthocyanin precursors: naringenin chalcone and naringenin) (Figure 2A,B; Table S2). In comparison to the pink stage fruit, nine differential flavonoids were present in the dark red stage fruit, of which six flavonoids were up-regulated (containing two major anthocyanins: cyanin chloride and peonidin 3-O-glucoside chloride), and three flavonoids were down-regulated (Figure 2A,B; Table S3). Contrastingly, 31 differentially expressed flavonoids appeared in the dark red stage fruit compared to the yellow stage fruit, with 14 up-regulated flavonoids (containing the 6 major anthocyanins: cyanidin 3-rutinoside, cyanidin 3-O-galactoside, cyanidin 3-O-glucoside, peonidin 3-O-glucoside chloride, cyanidin O-syringic acid, and cyanin chloride) and 17 down-regulated flavonoids (containing the anthocyanin precursor naringenin chalcone) (Figure 2A,B; Table S4).

### 2.2. Statistics of Transcriptome and lncRNA Data

Sweet cherry fruit ripening is accompanied by anthocyanin accumulation, indicated by the color change from the yellow stage to the dark red stage. To explore the functions of lncRNAs in sweet cherry fruit ripening, transcriptome and lncRNA sequencing were conducted on nine sweet cherry fruit libraries at yellow, pink, and dark red stages, with three biological replicates per stage. After data filtering, a total of 113.55, 114.69, 112.88, 133.52, 117.96, 114.27, 130.05, 114.40, and 109.79 million clean reads in samples of Y1 (yellow stage 1), Y2 (yellow stage 2), Y3 (yellow stage 3), P1 (pink stage 1), P2 (pink stage 2), P3 (pink stage 3), DR1 (dark red stage 1), DR2 (dark red stage 2) and DR3 (dark red stage 3) were generated, respectively (Table 1), and 90.09 (Y1), 91.60 (Y2), 96.36 (Y3), 106.04 (P1), 96.62 (P2), 94.32 (P3), 107.59 (DR1), 94.67 (DR2), and 90.26 (DR3) million reads were mapped to the sweet cherry reference genome. The mapping rates varied from 78.42% to 82.75% (Table 1).

### 2.3. Spatio-Temporal Specific Expression of Ripening-Associated Genes Drives Sweet Cherry Fruit Ripening

**Figure 3:**
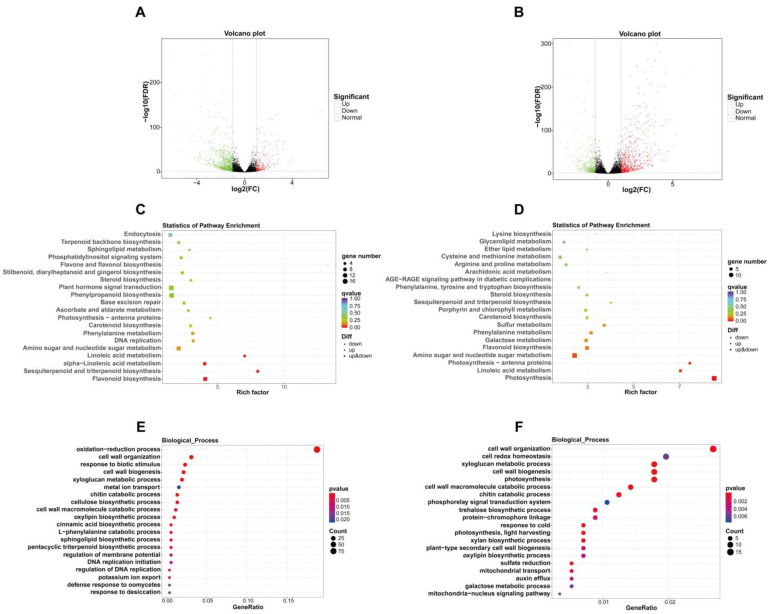
Analysis of DEGs in sweet cherry fruit at yellow stage, pink stage, and dark red stage. (**A**) Volcano plot of DEGs in sweet cherry fruit at yellow stage and pink stage. (**B**) Volcano plot of DEGs in sweet cherry fruit at pink stage and dark red stage. (**C**) KEGG analysis of DEGs in sweet cherry fruit at yellow stage and pink stage. (**D**) KEGG analysis of DEGs in sweet cherry fruit at pink stage and dark red stage. (**E**) GO analysis of DEGs in sweet cherry fruit at yellow stage and pink stage. (**F**) GO analysis of DEGs in sweet cherry fruit at pink stage and dark red stage. Rich factor: the ratio of the number of DEGs annotated in one pathway to the number of all genes annotated in that pathway. GeneRatio: the ratio of the number of DEGs annotated in one term to all DEG number. Qvalue is the correction of pvalue after multiple hypothesis testing. A smaller qvalue indicates a more reliable enrichment significance of DEGs in this pathway.

Transcriptome sequencing showed that 1150 genes were differentially expressed when sweet cherry fruit shifted from the yellow stage to the pink stage, of which 907 differentially expressed genes (DEGs) were down-regulated and 243 DEGs were up-regulated (Figure 3A; Table S5). Kyoto Encyclopedia of Genes and Genomes (KEGG) analysis exhibited that DEGs were enriched in the flavone and flavonol biosynthesis, plant hormone signal transduction, carotenoid biosynthesis, and flavonoid biosynthesis pathways, which are tightly related to fruit pigment buildup and phytohormone signaling during ripening (Figure 3C). Gene ontology (GO) analysis showed that DEGs were enriched in several biological processes related to cell wall metabolism, such as cell wall organization, cell wall biogenesis, the xyloglucan metabolic process, the cellulose biosynthetic process, and the cell wall macromolecule catabolic process, which are important metabolic processes during fruit ripening and softening (Figure 3E). Specifically, For ABA metabolism, the transcription of the ABA-hydrolyzing enzyme genes *abscisic acid 8’-hydroxylase 1-like* (*CYP707A1-like*), *CYP707A4-like1*, and *CYP707A4-like2* was down-regulated. In terms of cell wall metabolism, the transcription of cell wall-degrading enzyme genes *polygalacturonase QRT3* (*PG QRT3*) and *beta-galactosidase* (*TBG*) was up-regulated, along with decreased transcript levels of *pectin methylesterase (PME) inhibitor1/22/34*. In the anthocyanin production pathway, the expression of *chalcone synthase 1-like* (*CHS1-like*), *chalcone-flavonol isomerase 3* (*CHI3*) and *dihydroflavonol 4-reductase* (*DFR*) were elevated. In addition, several transcription factors that control sweet cherry fruit ripening were also differentially expressed, including *MYB10.1*, *basic helix-loop-helix 33* (*bHLH33*), *auxin response factor 8* (*ARF8*), and *DNA-binding with One Finger 15* (*Dof15*) (Table 2; Table S5).

In comparison to pink stage fruit, 1247 DEGs were identified in dark red stage fruit, among them 390 genes were down-regulated and 857 genes were up-regulated (Figure 3B; Table S6). KEGG analysis revealed that DEGs were enriched in pigment synthesis and cell wall polysaccharide metabolism pathways, including carotenoid biosynthesis, flavonoid biosynthesis, and galactose metabolism (Figure 3D). GO analysis displayed that DEGs were enriched in multiple biological processes in relation to cell wall metabolism, including cell wall organization, xyloglucan metabolic process, cell wall biogenesis, cell wall macromolecule catabolic process, plant-type secondary cell wall biogenesis, and galactose metabolic process (Figure 3F). To be specific, with regard to cell wall modification, the expression of *PG QRT3*, *PG-like*, *xyloglucan endotransglucosylase/hydrolase 2-like1* (*XTH2-like1*), *XTH2-like2*, *XTH2-like3*, *XTH8*, *XTH23*, *XTH24*, *XTH33*, *endoglucanase 9-like* (*CEL9-like*), *CEL CX*, *expansin-like A2.1* (*EXP-like A2.1*), and *EXP-like A2.2* were up-regulated, whereas the expression of *PG inhibitor 1-like*, *PME inhibitor2*, and *PME inhibitor34* were reduced. For the anthocyanin biosynthesis pathway, the transcript levels of *CHS1-like*, *CHI*, and *DFR* were raised. In addition, numerous ripening-associated MYB, bHLH, and WD40 family transcription factors were also differentially expressed (Table 3; Table S6).

The above results suggest that the distinct ripening stages of sweet cherry fruit are driven by the involvement of specific metabolic enzymes; moreover, the fine expression of transcription factors may execute essential regulatory functions during sweet cherry fruit ripening.

### 2.4. Identification of lncRNAs in Sweet Cherry Fruit

**Figure 4:**
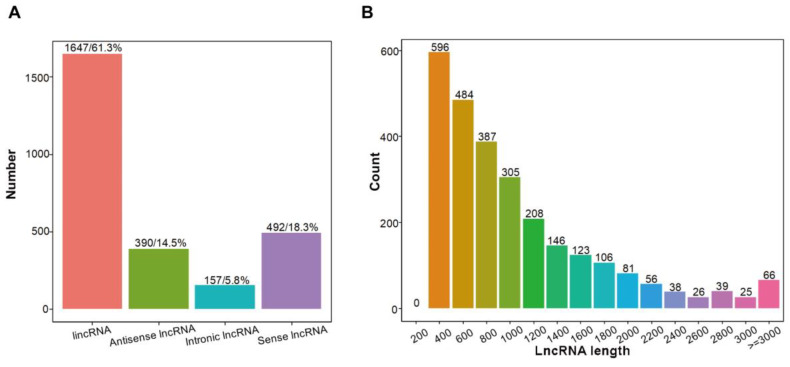
Characterization of lncRNAs in sweet cherry fruit at yellow stage, pink stage, and dark red stage. (**A**) Classification of lncRNAs identified in sweet cherry fruit at different ripening stages. (**B**) Length of lncRNAs identified in sweet cherry fruit at different ripening stages.

In total, 2686 lncRNAs were identified in cherry fruit samples at the yellow stage, pink stage and dark red stage by lncRNA sequencing, consisting of 1647 lincRNAs (61.3%), 390 antisense-lncRNA (14.5%), 157 intronic-lncRNA (5.8%), and 492 sense-lncRNA (18.3%) (Figure 4A; Table S7). LncRNAs varied in length from 202 nt to 10,491 nt, among which lncRNAs of 200–400 nt accounted for the largest proportion (22.34%) (Figure 4B; Table S7).

### 2.5. Differential Expression and Target Gene Enrichment of lncRNAs

**Figure 5:**
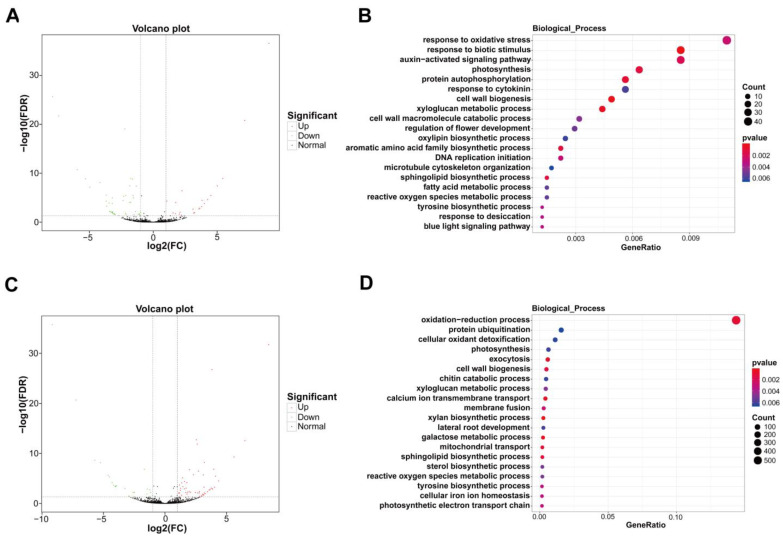
Analysis of DE-lncRNAs and their target genes in sweet cherry fruit at yellow stage, pink stage, and dark red stage. (**A**) Volcano plot of DE-lncRNAs in sweet cherry fruit at yellow stage and pink stage. (**B**) GO analysis of the target genes of DE-lncRNAs in sweet cherry fruit at yellow stage and pink stage. (**C**) Volcano plot of DE-lncRNAs in sweet cherry fruit at pink stage and dark red stage. (**D**) GO analysis of the target genes of DE-lncRNAs in sweet cherry fruit at pink stage and dark red stage. GeneRatio: the ratio of the number of target genes annotated in one term to all target gene numbers.

Compared with yellow stage fruit, a total of 66 lncRNAs in pink stage fruit showed differential expression, among which 44 differentially expressed lncRNAs (DE-lncRNAs) were reduced and 22 DE-lncRNAs were elevated (Figure 5A; Table S8). GO analysis revealed that the target genes of these DE-lncRNAs were enriched in biological processes associated with cell wall metabolism, including cell wall biogenesis, the xyloglucan metabolic process, and the cell wall macromolecule catabolic process (Figure 5B).

In comparison to pink stage fruit, 79 DE-lncRNAs appeared in dark red stage fruit, among which 25 lncRNAs were decreased and 54 lncRNAs were raised (Figure 5C; Table S9). GO analysis showed that the target genes of these DE-lncRNAs were enriched in biological processes of cell wall biogenesis, the xyloglucan metabolic process, and the galactose metabolic process, which are tightly correlated with cell wall degradation during fruit ripening and softening (Figure 5D).

### 2.6. Verification of Transcriptome and lncRNA Data

**Figure 6:**
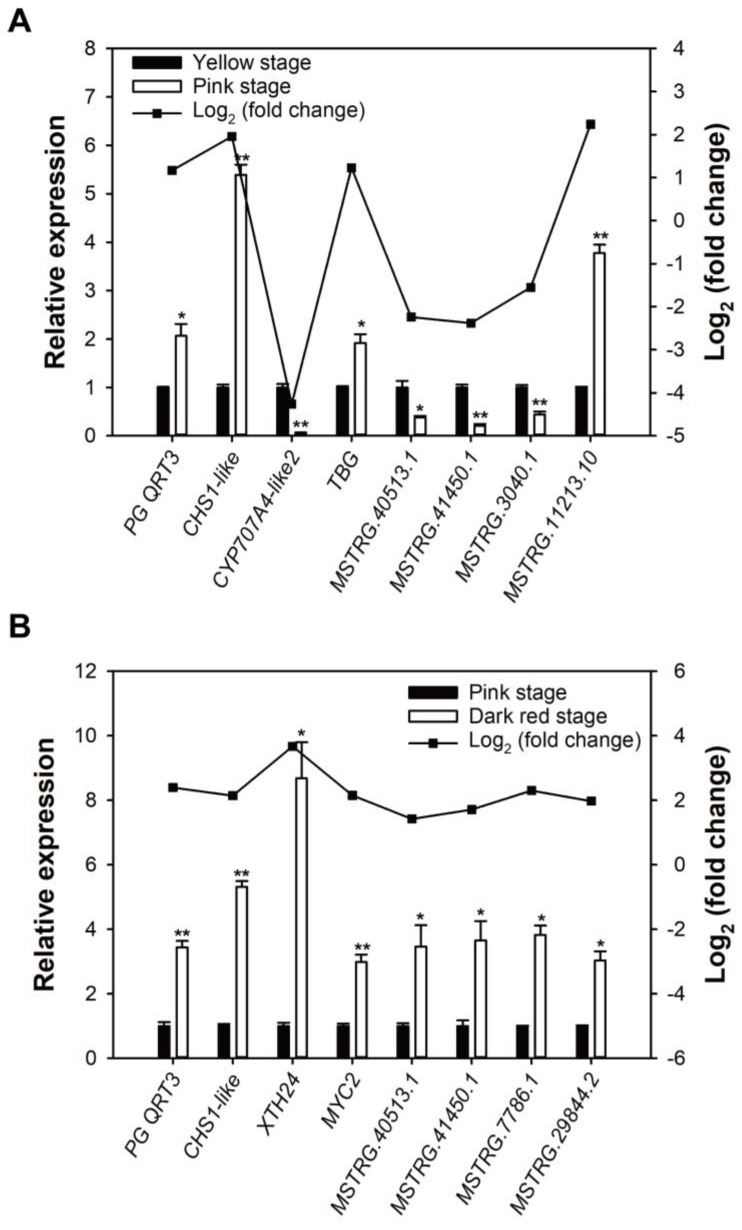
RT-qPCR verification of the results of transcriptome and lncRNA sequencing. (**A**) The relative expression and fold change of DEGs and DE-lncRNAs in sweet cherry fruit at yellow stage and pink stage. (**B**) The relative expression and fold change of DEGs and DE-lncRNAs in sweet cherry fruit at pink stage and dark red stage. Error bars represent the SE (n = 3). Asterisk denotes significant difference between two groups (* *p* < 0.05, ** *p* < 0.01).

To examine the dependability of the transcriptome and lncRNA sequencing results, eight ripening-related DEGs and DE-lncRNAs were selected according to the comparative transcriptomes of yellow stage vs. pink stage, and pink stage vs. dark red stage, respectively, for real-time quantitative PCR (RT-qPCR) validation. The RT-qPCR results show that these DEGs and DE-lncRNAs displayed coincident change trends with the sequencing results of transcriptome and lncRNA (Figure 6A,B), which verified that the sequencing data were reliable.

### 2.7. lncRNAs Were Involved in the Ripening Process of Sweet Cherry Fruit

To further elucidate the function of lncRNAs in sweet cherry fruit ripening, we performed an integrated analysis for DEGs and DE-lncRNAs and found that many ripening-associated DEGs could serve as target genes of DE-lncRNAs (Table 2 and Table 3 and Tables S10 and S11).

When fruit transition from the yellow stage to the pink stage, most ripening-related DEGs can be targeted by DE-lncRNAs except for *CHS1-like* (Table 2). For instance, the transcription level of lncRNA *MSTRG.3112.1* and *MSTRG.37034.2* were down-regulated, and the expression level of their target gene *CYP707A1-like* (encoding ABA-degrading enzyme) was also down-regulated. *TBG*, a gene-encoding cell wall-degrading enzyme with up-regulated expression level, could serve as the target of five DE-lncRNAs, in which the expression of *MSTRG.30065.2*, *MSTRG.35294.2*, and *MSTRG.38400.3* were positively correlated with that of *TBG*, while the expression of *MSTRG.3112.1* and *MSTRG.9626.1* exhibited a negative correlation with *TBG*. The expression of *MSTRG.12848.1* and *MSTRG.29836.1* were up-regulated, as well as that of its target gene *DFR* (a key gene for anthocyanin biosynthesis). The differentially expressed ripening-associated transcription factor genes *MYB10.1*, *bHLH33*, *ARF8*, and *Dof15* could be targeted by 3, 8, 11, and 12 DE-lncRNAs, respectively, and the transcription levels of these DE-lncRNAs showed a positive or negative correlation with that of their targets (Table 2).

Compared with pink stage fruit, most of the ripening-associated DEGs in dark red stage fruit could also act as targets of DE-lncRNAs (Table 3). For instance, many XTH-encoding genes with up-regulated expression (*XTH2-like1*, *XTH2-like2*, *XTH2-like3*, *XTH8*, *XTH23*, *XTH24*, and *XTH33*) were subjected to the regulation of upstream DE-lncRNAs. The key genes for anthocyanin biosynthesis, *CHS1-like*, *CHI* and *DFR* can act as the downstream targets of 10, 10, and 9 DE-lncRNAs, respectively. Furthermore, a series of differentially expressed *MYB*, *bHLH*, and *WD40* family transcription factors can be regulated by lncRNAs as downstream targets, and their transcriptional levels showed a correlation with that of their upstream lncRNAs (Table 3).

Overall, during sweet cherry fruit ripening, ripening-related DEGs could be targeted by 67 DE-lncRNAs, including 48 lincRNAs (71.6%), 12 sense lncRNAs (17.9%), 5 antisense lncRNAs (7.5%), and 2 intronic lncRNAs (3.0%) (Table 2 and Table 3 and Table S7), which indicated that lncRNAs (dominated by lincRNAs) can modulate the ripening process of sweet cherry fruit by targeting ripening-related metabolic enzyme or transcription factor genes.

### 2.8. Pearson’s Correlation Analysis of Anthocyanin Metabolites and Associated DEGs/DE-lncRNAs

In order to further determine the correlation between the accumulation levels of major anthocyanins (cyanidin 3-rutinoside, cyanidin 3-O-galactoside, cyanidin 3-O-glucoside, peonidin 3-O-glucoside chloride, cyanidin O-syringic acid, and cyanin chloride) and the expression levels of related DEGs/DE-lncRNAs during sweet cherry fruit ripening, Pearson’s correlation analysis was performed on the abundance of major anthocyanins and the expression levels (fragments per kilobase of script per million fragments mapped (FPKM)) of related DEGs/DE-lncRNAs in sweet cherry fruit at different ripening stages. The results show that, in terms of metabolic enzyme genes, the expression levels of ABA-degrading genes *CYP707A4-like1* and *CYP707A4-like2* were significantly negatively correlated with the abundance of major anthocyanins. The expression levels of the anthocyanin biosynthesis genes *CHS-like*, *CHI*, *CHI3* and *DFR* showed a significant positive correlation with the abundance of major anthocyanins (Table 4). As for transcription factor genes, the expression levels of *MYB10.1*, *MYB44-like*, *MYB10 V1-3*, *bHLH113*, and *MYC2* exhibited a significant positive correlation with the major anthocyanin levels, whereas the expression levels of *bHLH33*, *bHLH51*, and *bHLH92* displayed a significant negative correlation with the abundance of major anthocyanins (Table 4). Regarding lncRNAs, the transcript levels of 45 lncRNAs presented a significant correlation with the abundance of major anthocyanins, of which 30 lncRNAs showed a positive correlation and 15 lncRNAs showed a negative correlation (Table 4).

## 3. Discussion

Sweet cherry belongs to non-climacteric fruit with general ripening characteristics of fleshy fruit [3]. LncRNAs are a class of regulators involved in various plant biological processes [9]. In this study, we conducted widely targeted metabolomics, transcriptomics and lncRNA analyses of sweet cherry fruit at different ripening stages (yellow stage, pink stage, and dark red stage) and further revealed the potential molecular mechanisms of sweet cherry fruit ripening by integrated analysis.

Widely targeted metabolomics analysis showed that a considerable number of metabolic changes occurred throughout sweet cherry fruit ripening, and the number of differential metabolites gradually increased with the ripening process, among which the increased anthocyanin content in flavonoids were directly correlated with the pigment accumulation in sweet cherry fruit. As the most abundant anthocyanins in sweet cherry fruit, cyanidin 3-rutinoside, cyanidin 3-O-galactoside, and cyanidin 3-O-glucoside accumulated rapidly during ripening, whereas the anthocyanin precursor naringenin chalcone and naringenin showed a decreasing trend, which suggests that the conversion of naringenin chalcone and naringenin to cyanidin-glycosides is the primary pathway for the buildup of anthocyanins in sweet cherry fruit.

Compared to yellow stage fruit, KEGG and GO analyses showed that DEGs in pink stage fruit were enriched in pathways and biological processes associated with phytohormones, pigment synthesis and cell wall metabolism, including plant hormone signal transduction, flavone and flavonol biosynthesis, carotenoid biosynthesis, flavonoid biosynthesis pathways, cell wall organization, cell wall biogenesis, the xyloglucan metabolic process, the cellulose biosynthetic process, and the cell wall macromolecule catabolic process. ABA is the dominant phytohormone that facilitates sweet cherry fruit ripening and accumulates to a high level during fruit ripening [34]. 9-cis-epoxycarotenoid dioxygenases (NCEDs) are the core enzymes for ABA synthesis, which convert zeaxanthin into ABA, whereas the CYP707A subfamily are the pivotal enzymes mediating the ABA decomposition pathway, catalyzing the hydroxylation of ABA into hydroxy ABA. Dynamic changes of ABA content in plants are controlled by the expression levels of *NCED* and *CYP707A* [35]. In these DEGs, the expression of *CYP707A1-like*, *CYP707A4-like1*, and *CYP707A4-like2* were down-regulated, suggesting that they may be important metabolic enzymes that promote ABA accumulation as sweet cherry fruit ripens. Sweet cherry fruit ripening is coupled with fruit softening, which is induced by the action of a series of cell wall-degrading enzymes [3]. Pectin is a key component in cell walls. PG is responsible for the degradation of polygalacturonic acid in pectin. TBG can remove galactose residues from pectin. PME can facilitate further degradation of pectin by reducing the degree of methyl esterification in pectin [36], and PME inhibitor can suppress the activity of PME [37]. During the transformation from the yellow stage to the pink stage of sweet cherry fruit, the expression of *PG QRT3* and *TBG* were enhanced, whereas the expression of *PME inhibitor1*/*22*/*34* was suppressed, which may be the essential molecular dynamics changes for cell wall degradation during the early fruit ripening. Another remarkable metabolic alteration when sweet cherry fruit ripens is the rapid anthocyanin accumulation [34]. In the pathway of anthocyanin biosynthesis, CHS contributes to the condensation of malonyl-coenzyme A (CoA) and 4-coumaroyl-CoA into naringenin chalcone, which is converted to naringenin via the catalysis of CHI. Naringenin further forms dihydroflavonols in the presence of flavanone 3-hydroxylase (F3H). Subsequently, DFR can convert dihydroflavonols to leucoanthocyanins, which can be transformed into multiple forms of anthocyanins under the action of anthocyanidin synthase (ANS) and uridine diphosphate-glucose:flavonoid 3-O-glucosyltransferase (UFGT) [38]. The increased transcript levels of *CHS1-like*, *CHI3*, and *DFR* were observed in pink stage fruit, accounting for the decrease in abundance of the anthocyanin precursor naringenin chalcone and naringenin, as well as the accumulation of six major anthocyanins from the yellow stage to the pink stage. Transcription factors also exert vital regulatory functions in sweet cherry fruit ripening. For instance, *MYB10.1* has been proven to be a key control locus for anthocyanin accumulation, positively regulating anthocyanin accumulation by targeting and activating *ANS* and *UFGT* [39], while bHLH33 is an inhibitor of anthocyanin production [40]. Dof15 can interact with the promoter of cell wall-degrading enzyme genes and inhibit their expression in sweet cherry fruit, while ARF8 can inhibit fruit softening by directly stimulating the expression of *Dof15* [41]. In pink stage fruit, the expression of *MYB10.1* was raised, while the expression of *bHLH33*, *ARF8*, and *Dof15* were decreased, suggesting that the fine expression of *MYB10.1*, *bHLH33*, *ARF8*, and *Dof15* perform key regulatory functions in the softening and anthocyanin accumulation during the early ripening periods of sweet cherry fruit. Additionally, the targets of DE-lncRNAs are enriched in biological processes in relation to cell wall metabolism, most of the ripening-associated DEGs can be targeted by DE-lncRNAs, and the same DE-lncRNA may target multiple ripening-associated DEGs. For example, lncRNA MSTRG.11213.10 can simultaneously target *CYP707A4-like1*, *CYP707A4-like2*, *PME inhibitor22*, *PME inhibitor34*, *bHLH33*, *ARF8* and *Dof15*, and another lncRNA MSTRG.26521.2 can simultaneously target *CYP707A4-like1*, *CYP707A4-like2*, *PME inhibitor34*, *MYB10.1*, *bHLH33* and *ARF8*, which suggests that lncRNAs can affect sweet cherry fruit ripening by targeting ripening-related DEGs, and the same lncRNA can regulate fruit ripening at different dimensions, e.g., lncRNA MSTRG.11213.10 and MSTRG.26521.2 can coordinate ABA metabolism, cell wall modification, anthocyanin production, and the fine expression of transcription factor genes to modulate fruit ripening.

In comparison to pink stage fruit, KEGG and GO analyses indicated that DEGs in dark red stage fruit were enriched in pathways and biological processes in relation to pigment synthesis and cell wall metabolism, including carotenoid biosynthesis, flavonoid biosynthesis, galactose metabolism, cell wall organization, xyloglucan metabolic process, cell wall biogenesis, cell wall macromolecule catabolic process, plant-type secondary cell wall biogenesis, and galactose metabolic process. In terms of cell wall metabolism, the transcription levels of many cell wall-degrading genes were altered, including *PG QRT3*, *PG-like*, *XTH2-like1*, *XTH2-like2*, *XTH2-like3*, *XTH8*, *XTH23*, *XTH24*, *XTH33*, *CEL9-like*, *CEL CX*, *EXP-like A2.1* and *EXP-like A2.2* with elevated expression, as well as *PG inhibitor 1-like*, *PME inhibitor2* and *PME inhibitor34* with decreased expression. Among such genes, XTH can promote the depolymerizing of hemicellulose in cell wall. CEL plays an essential role in degrading the cellulose–hemicellulose structure of cell wall. EXP is considered to relax the cell wall structure [42]. PG inhibitor can restrict the activity of PG [43]. In the anthocyanin production pathway, *CHS1-like*, *CHI*, and *DFR* expression levels were elevated, promoting further buildup of anthocyanins in sweet cherry fruit from the pink stage to dark red stage, particularly for cyanin chloride and peonidin 3-O-glucoside chloride. Moreover, many MYB, bHLH and WD40 family transcription factor genes are differentially expressed. For example, the expression levels of *bHLH13*, *bHLH74* and *MYC2* of bHLH family are up-regulated, among which bHLH13 and bHLH74 can activate the promoter of *ANS* [44], and MYC2 is implicated in the formation of anthocyanin precursors via activating *phenylalanine ammonia-lyase* (*PAL*) and *cinnamate-4-hydroxylase* (*C4H*) [45]. It was reported that MYB, bHLH and WD40 family transcription factors can elaborate the MYB-bHLH-WD40 complex to synergistically control fruit anthocyanin production [39]; therefore, these differentially expressed *MYB*, *bHLH* and *WD40* transcription factors may be prospective moderators during the anthocyanin accumulation of sweet cherry fruit. In addition, target gene prediction of DE-lncRNAs revealed that most of the ripening-related DEGs could also serve as target genes for DE-lncRNAs, and lncRNAs could be involved in fruit ripening regulation through different aspects. For example, lncRNA MSTRG.21139.1 can simultaneously target 14 ripening-associated DEGs, including *PG QRT3*, *PG-like*, *XTH2-like1*, *XTH2-like2*, *XTH2-like3*, *CEL CX*, *PME inhibitor2*, *CHS1-like*, *CHI*, *DFR*, *MYB44-like*, *bHLH51*, *bHLH113* and *MYC2*. lncRNA MSTRG.13122 can simultaneously target *PG QRT3*, *PG-like*, *XTH2-like2*, *CEL CX*, *PME inhibitor2*, *CHS1-like*, *CHI*, *DFR*, *MYB10 V1-3* and *bHLH51*, which participate in cell wall metabolism, anthocyanin accumulation and transcriptional regulation.

Throughout the entire ripening process from the yellow stage to the dark red stage, we found that the transcription level of cell wall degradation gene *PG QRT3*, as well as anthocyanin production genes, *CHS1-like* and *DFR*, increased continuously, while the transcript level of *PME inhibitor34* declined constantly, which indicated that these genes exerted important functions during the whole ripening process of sweet cherry fruit. For the upstream DE-lncRNAs of ripening-related DEGs, more than 70% of them belong to lincRNAs. LincRNAs are a class of lncRNAs located between two coding genes, which can regulate plant growth and development through a variety of action mechanisms, including participation in chromatin remodeling, transcriptional regulation, miRNA competition, and alternative splicing [9]. The high proportion of lincRNAs in DE-lncRNAs suggests that the dominant lncRNAs in the regulation of sweet cherry fruit ripening are lincRNAs. In addition, for the accumulation and regulation of anthocyanins, the Pearson’s correlation analysis showed that the expression levels of differential expressed key metabolic enzyme genes (*CYP707A4-like1*, *CYP707A4-like2*, *CHS-like*, *CHI*, *CHI3*, and *DFR*) and the transcription factor genes (*MYB10.1*, *MYB44-like*, *MYB10 V1-3*, *bHLH113*, *MYC2*, *bHLH33*, *bHLH51*, and *bHLH92*) were significantly correlated with the abundance of major anthocyanins. Also, the transcript levels of most DE-lncRNAs targeting DEGs related to anthocyanin biosynthesis and regulation showed significant correlation with the levels of major anthocyanins. For instance, MSTRG.13122.1, which can simultaneously target *CHS1-like*, *CHI*, *DFR*, *MYB10 V1-3*, and *bHLH51*, exhibited a significant positive correlation with the abundance of all six major anthocyanins, suggesting that the regulation of anthocyanin synthesis during sweet cherry fruit ripening was mediated by lncRNAs.

## 4. Materials and Methods

### 4.1. Fruit Material

Sweet cherry fruit (*Prunus avium* L. cv. Tieton) free of mechanical injury, pests, and diseases at yellow stage, pink stage, and dark red stage (Figure 1A) were harvested from Beijing Academy of Agriculture and Forestry Sciences and immediately transported to the lab within 1 h. The pitted fruit were chopped, rapidly frozen in liquid N_2_, and kept at −80 °C for further analysis. The samples per period were divided into three sets of biological replicates, with 30 fruit in each biological replicate.

### 4.2. Widely Targeted Metabolomics Detection

Sweet cherry fruit samples at different ripening stages (yellow stage, pink stage, and dark red stage) were submitted to Wuhan Metware Biotechnology Co., Ltd. (Wuhan, China; www.metware.cn, accessed on 10 May 2024) for widely targeted metabolomics analysis.

Sample preparation and extraction: the sweet cherry fruit at different ripening stages were freeze-dried and crushed into powder. Then, 100 mg of powder was taken and dissolved in 0.6 mL of 70% methanol, then extracted at 4 °C for 12–16 h (during this period it was vortexed six times), followed by a centrifugation (10,000× *g* for 10 min), and the supernatant was filtered via a 0.22 μm micropore filter and kept in sample vials for ultra-performance liquid chromatography-tandem mass spectrometry (UPLC-MS/MS) analysis.

UPLC-MS/MS parameters: 4 µL extracts were injected into the UPLC-MS/MS system (Shim-pack UFLC SHIMADZU CBM30A; Applied Biosystems 4500 QTRAP) with ACQUITY UPLC HSS T3 C18 column (1.8 µm, 2.1 × 100 mm, Waters). The mobile phase A is ultra-pure water (adding 0.04% acetic acid), and mobile phase B is acetonitrile (adding 0.04% acetic acid). Elution gradient for mobile phase B is 5% at 0.00 min, which is then increased linearly to 95% within 10.00 min, maintained for 1 min, decreased to 5% from 11.00 to 11.10 min, and balanced at 5% to 14.00 min. The flow rate was 0.35 mL min^−1^. The temperature of column was programmed at 40 °C. The temperature of electrospray ionization was 550 °C. The voltage for the mass spectrometry was 5500 V. The curtain gas was set at 30 psi. The parameter of collision-activated dissociation was set as high. In the case of triple quadrupole MS, each ion pair was scanning identified according to the optimal declustering potential and collision energy.

Metabolite qualification is based on secondary profiling information from the self-constructed metware database. Metabolite relative quantitation was achieved using the multiple reaction monitoring of MS and corrected peak area. Principal component analysis (PCA) was conducted with statistics function prcomp within R software 3.5.0 (www.r-project.org/, accessed on 12 June 2024) after data was unit variance scaled. Orthogonal partial least squares-discriminant analysis (OPLS-DA) was performed by MetaboAnalystR 1.0.1 in R package following the log_2_ transform and mean centering for data to generate the values of variable importance in project (VIP) for differential metabolite screen, with a permutation test (200 permutations) to avoid overfitting. The filtering criterion for differential metabolites was |log_2_ (fold change)| > 1, and VIP values ≥ 1.

### 4.3. Transcriptome and lncRNA Sequencing

Transcriptome and lncRNA sequencing of sweet cherry fruit at yellow stage, pink stage, and dark red stage was carried out by Biomarker Technologies Co., Ltd. (Beijing, China; www.biomarker.com.cn, accessed on 10 May 2024), based on the Illumina sequencing platform (NovaSeq 6000) with a 50× sequencing depth. The bioinformatics analyses were carried out with the BMKCloud (https://www.biocloud.net/, accessed on 12 June 2024).

The Raw Data output from the sequencing platform was filtered (excluding low-quality, adapter, and uncertain reads) to get clean reads, which were mapped to the sweet cherry reference genome (v1.0.a1.Prunus_avium.v1.0.a1.genome.fa) by hierarchical indexing for spliced alignment of transcripts (HISAT 2.0.4), with mapping rate as the ratio of mapped reads to clean reads. StringTie 1.3.1 was used to further assemble and quantify the mapped reads [46,47].

The identification of lncRNAs included basic and coding capacity screening. Transcripts in the basic screening process should meet the following conditions: (i) class_code is “i”, “x”, “u”, “o”, or “e” (ii) length ≥ 200 bp, and exon number ≥ 2 (iii) FPKM ≥ 0.1 [48,49]. Candidate lncRNAs that passed the basic screening were then subjected to a coding capacity screening to exclude the transcripts with coding capacity. The coding capacity screening was based on coding potential calculator (CPC) analysis [50], coding-non-coding index (CNCI) analysis [51], coding potential assessment tool (CPAT) analysis [52], and Pfam protein structural domain analysis [53], and the final lncRNAs identified in the samples were the intersection of transcripts that passed the analysis of CPC, CNCI, CPAT, and Pfam, respectively.

The target gene prediction of lncRNAs was performed in accordance with the location-relation prediction and expression correlation prediction. For location-relation prediction, Perl script was utilized to predict neighboring genes of lncRNA inside 100 kilobase (kb) as its *cis*-target genes. For expression correlation prediction, Pearson correlation coefficient (PCC) manner was employed to examine the relevance of lncRNA and mRNA in samples, and genes whose |PCC| > 0.9 and significance *p*-value < 0.01 were predicted as the *trans*-target genes for lncRNA.

FPKM was employed to evaluate the transcript levels of mRNAs and lncRNAs, and DESeq2 was used to screen the DEGs and DE-lncRNAs [54]. The judging criterion for DEGs and DE-lncRNAs were fold change of FPKM ≥ 2 and false discovery rate (FDR) < 0.05. KEGG annotation and pathway enrichment analysis of DEGs were referred to the website (http://www.genome.ad.jp/kegg/, accessed on 12 June 2024) [55]. GO enrichment analysis of DEGs was carried out based on ClusterProfiler (http://bioconductor.org/packages/release/bioc/html/clusterProfiler.html, accessed on 12 June 2024) [56,57].

### 4.4. RT-qPCR

Total RNA of sweet cherry fruit was isolated by FastPure Plant Total RNA Isolation Kit (Polysaccharides & Polyphenolics-rich) (Vazyme Biotech Co., Ltd., Nanjing, China), and 1 μg of total RNA was reverse transcribed into cDNA by HiScript^®^ III All-in-one RT SuperMix Perfect (Vazyme Biotech Co., Ltd., Nanjing, China). The RT-qPCR reaction system was prepared with ChamQ SYBR qPCR Master Mix (Vazyme Biotech Co., Ltd., Nanjing, China) and run on a CFX96 Real-Time PCR System (Bio-Rad, Hercules, CA, USA). The *Actin* gene was chosen as an internal reference gene for normalization. The relative expression of mRNAs or lncRNAs was computed via the 2^−ΔΔCt^ manner [58] based on three sets of biological replicates. Primers were shown in Table S12.

### 4.5. Statistical Analysis

RT-qPCR results were presented as mean ± standard error (SE) (n = 3) and subjected to independent sample *T*-test by IBM SPSS statistics 22 software. Asterisk denotes significant difference between two groups (* *p* < 0.05, ** *p* < 0.01). Pearson’s correlation analysis was performed by IBM SPSS statistics 22 software. Metabolite heatmap was generated via BMKCloud (https://www.biocloud.net/, accessed on 12 June 2024). Venn diagram was drawing by OmicShare Tools (https://www.omicshare.com/tools/, accessed on 12 June 2024).

## 5. Conclusions

**Figure 7:**
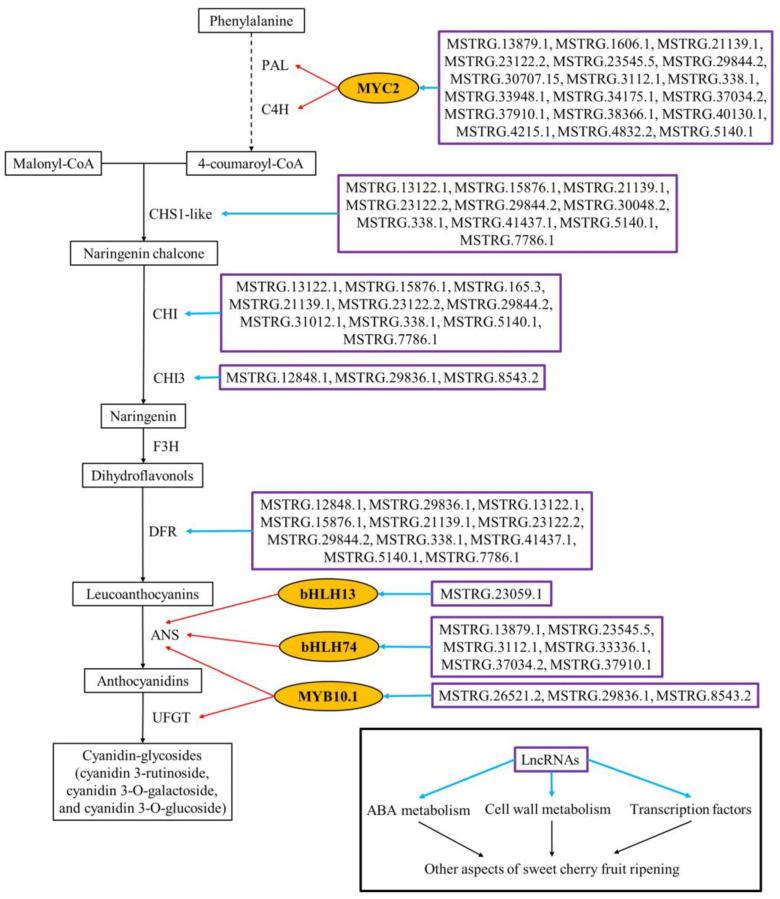
Regulatory network of anthocyanin biosynthesis and other ripening aspects mediated by lncRNAs in sweet cherry fruit. The dotted line represents a multi-step reaction. The red arrows represent transcriptional activation. The yellow ovals represent transcription factors. Purple boxes represent upstream DE-lncRNAs of target genes.

In conclusion, this study outlined the metabolic alterations and the expression patterns of mRNAs and lncRNAs of sweet cherry fruit at different ripening stages (the yellow stage, pink stage, and dark red stage) and revealed the potential molecular mechanisms of sweet cherry fruit ripening from the lncRNA-mRNA perspective through the differential expression analysis and target gene prediction of lncRNAs, discovering that lncRNAs can regulate sweet cherry fruit ripening in a multidimensional manner by targeting the genes of ABA metabolism, cell wall modification, anthocyanin production, and transcription factors (Figure 7).

## Figures and Tables

**Table 1:** Output results and mapping rate of transcriptome and lncRNA sequencing.

Samples	Clean Reads (million)	Mapped Reads (million)	Mapping Rate
Y1	113.55	90.09	79.34%
Y2	114.69	91.6	79.87%
Y3	112.88	96.36	78.42%
P1	133.52	106.04	79.42%
P2	117.96	96.62	81.91%
P3	114.27	94.32	82.54%
DR1	130.05	107.59	82.72%
DR2	114.4	94.67	82.75%
DR3	109.79	90.26	82.21%

**Table 2:** DE-lncRNAs and their targets for ripening-related DEGs between sweet cherry fruit at yellow stage and pink stage.

Gene ID	Log_2_ (Fold Change)	Annotation	Upstream DE-lncRNAs
Pav_sc0001440.1_g080.1.mk	−2.08	CYP707A1-like	MSTRG.3112.1 (−1.85), MSTRG.37034.2 (−1.86)
Pav_sc0000852.1_g900.1.mk	−2.50	CYP707A4-like1	MSTRG.10166.2 (2.03), MSTRG.11213.10 (2.24), MSTRG.26521.2 (−2.33), MSTRG.3040.1 (−1.55), MSTRG.30960.1 (−3.27), MSTRG.34331.1 (−3.22), MSTRG.36057.7 (−3.69), MSTRG.36335.1 (−2.14), MSTRG.40513.1 (−2.24), MSTRG.8543.2 (−1.07)
Pav_sc0000071.1_g720.1.mk	−4.26	CYP707A4-like2	MSTRG.11213.10 (2.24), MSTRG.26521.2 (−2.33), MSTRG.3040.1 (−1.55), MSTRG.30960.1 (−3.27), MSTRG.34331.1 (−3.22), MSTRG.36335.1 (−2.14), MSTRG.8543.2 (−1.07)
Pav_sc0000020.1_g060.1.mk	1.17	PG QRT3	MSTRG.12848.1 (2.08), MSTRG.37034.2 (−1.86)
Pav_sc0000212.1_g1900.1.mk	1.23	TBG	MSTRG.30065.2 (7.15), MSTRG.3112.1 (−1.85), MSTRG.35294.2 (4.50), MSTRG.38400.3 (3.10), MSTRG.9626.1 (−3.69)
Pav_sc0000028.1_g320.1.mk	−1.99	PME inhibitor1	MSTRG.3112.1 (−1.85), MSTRG.37034.2 (−1.86)
Pav_sc0004475.1_g020.1.br	−1.10	PME inhibitor22	MSTRG.18052.1 (−1.40), MSTRG.2169.2 (−1.59), MSTRG.25930.8 (−3.20), MSTRG.2981.10 (−1.62), MSTRG.30065.2 (7.15), MSTRG.33128.1 (−1.77), MSTRG.33241.2 (−1.18), MSTRG.35294.2 (4.50), MSTRG.37117.4 (5.00), MSTRG.38400.3 (3.10), MSTRG.40513.1 (−2.24), MSTRG.41450.1 (−2.38)
Pav_sc0001335.1_g240.1.mk	−1.38	PME inhibitor34	MSTRG.10166.2 (2.03), MSTRG.11213.10 (2.24), MSTRG.26521.2 (−2.33), MSTRG.2981.4 (−1.01), MSTRG.29836.1 (4.25), MSTRG.30960.1 (−3.27), MSTRG.33370.3 (−1.1), MSTRG.34331.1 (−3.22), MSTRG.8543.2 (−1.07)
Pav_sc0000045.1_g280.1.mk	1.96	CHS1-like	
Pav_sc0005746.1_g030.1.mk	1.83	CHI3	MSTRG.12848.1 (2.08), MSTRG.29836.1 (4.25), MSTRG.8543.2 (−1.07)
Pav_sc0002208.1_g840.1.mk	2.16	DFR	MSTRG.12848.1 (2.08), MSTRG.29836.1 (4.25)
Pav_sc0000464.1_g130.1.br	1.97	MYB10.1	MSTRG.26521.2 (−2.33), MSTRG.29836.1 (4.25), MSTRG.8543.2 (−1.07)
Pav_sc0000102.1_g430.1.mk	−1.39	bHLH33	MSTRG.10166.2 (2.03), MSTRG.11213.10 (2.24), MSTRG.13193.1 (1.06), MSTRG.26521.2 (−2.33), MSTRG.3040.1 (−1.55), MSTRG.30960.1 (−3.27), MSTRG.33370.3 (−1.10), MSTRG.8543.2 (−1.07)
Pav_sc0001900.1_g150.1.mk	−1.34	ARF8	MSTRG.10166.2 (2.03), MSTRG.11213.10 (2.24), MSTRG.26521.2 (−2.33), MSTRG.2981.4 (−1.01), MSTRG.29836.1 (4.25), MSTRG.3040.1 (−1.55), MSTRG.30960.1 (−3.27), MSTRG.33370.3 (−1.10), MSTRG.34331.1 (−3.22), MSTRG.36057.7 (−3.69), MSTRG.8543.2 (−1.07)
Pav_sc0000358.1_g850.1.mk	−1.39	Dof15	MSTRG.18052.1 (−1.40), MSTRG.2169.2 (−1.59), MSTRG.25930.8 (−3.20), MSTRG.2642.1 (5.43), MSTRG.2981.10 (−1.62), MSTRG.30065.2 (7.15), MSTRG.33128.1 (−1.77), MSTRG.33241.2 (−1.18), MSTRG.35294.2 (4.50), MSTRG.37117.4 (5.00), MSTRG.38400.3 (3.10), MSTRG.40513.1 (−2.24)

The number following DE-lncRNA represents the value of log_2_ (fold change of this lncRNA).

**Table 3:** DE-lncRNAs and their targets for ripening-related DEGs between sweet cherry fruit at pink stage and dark red stage.

Gene ID	Log_2_ (Fold Change)	Annotation	Upstream DE-lncRNAs
Pav_sc0000020.1_g060.1.mk	2.39	PG QRT3	MSTRG.13122.1 (1.26), MSTRG.15876.1 (−1.69), MSTRG.165.3 (1.84), MSTRG.21139.1 (3.20), MSTRG.22209.11 (−4.63), MSTRG.23122.2 (1.24), MSTRG.23545.5 (2.47), MSTRG.29844.2 (1.97), MSTRG.30707.15 (−5.26), MSTRG.31012.1 (2.79), MSTRG.338.1 (3.11), MSTRG.33948.1 (1.57), MSTRG.37034.2 (3.80), MSTRG.40130.1 (2.53), MSTRG.41437.1 (1.45), MSTRG.4832.2 (4.35), MSTRG.5140.1 (8.41), MSTRG.7786.1 (2.30)
Pav_sc0003823.1_g150.1.mk	1.09	PG-like	MSTRG.13122.1 (1.26), MSTRG.15876.1 (−1.69), MSTRG.21139.1 (3.20), MSTRG.23122.2 (1.24), MSTRG.338.1 (3.11), MSTRG.41437.1 (1.45), MSTRG.5140.1 (8.41), MSTRG.7786.1 (2.30)
Pav_sc0000428.1_g510.1.mk	3.29	XTH2-like1	MSTRG.15876.1 (−1.69), MSTRG.1606.1 (1.57), MSTRG.21139.1 (3.20), MSTRG.23122.2 (1.24), MSTRG.30707.15 (−5.26), MSTRG.3112.1 (2.62), MSTRG.338.1 (3.11), MSTRG.33948.1 (1.57), MSTRG.34175.1 (1.78), MSTRG.35257.14 (6.49), MSTRG.37034.2 (3.80), MSTRG.37910.1 (2.72), MSTRG.38366.1 (3.16), MSTRG.40130.1 (2.53), MSTRG.41437.1 (1.45), MSTRG.4215.1 (1.35), MSTRG.4832.2 (4.35), MSTRG.5140.1 (8.41)
Pav_sc0000428.1_g520.1.mk	2.49	XTH2-like2	MSTRG.13122.1 (1.26), MSTRG.15876.1 (−1.69), MSTRG.1606.1 (1.57), MSTRG.21139.1 (3.20), MSTRG.23122.2 (1.24), MSTRG.29844.2 (1.97), MSTRG.30536.1 (3.65), MSTRG.30707.15 (−5.26), MSTRG.31012.1 (2.79), MSTRG.338.1 (3.11), MSTRG.33948.1 (1.57), MSTRG.34175.1 (1.78), MSTRG.37034.2 (3.80), MSTRG.37910.1 (2.72), MSTRG.38366.1 (3.16), MSTRG.40130.1 (2.53), MSTRG.41437.1 (1.45), MSTRG.4215.1 (1.35), MSTRG.4832.2 (4.35), MSTRG.5140.1 (8.41), MSTRG.7786.1 (2.30)
Pav_sc0000428.1_g530.1.mk	3.61	XTH2-like3	MSTRG.15876.1 (−1.69), MSTRG.1606.1 (1.57), MSTRG.21139.1 (3.20), MSTRG.30707.15 (−5.26), MSTRG.3112.1 (2.62), MSTRG.338.1 (3.11), MSTRG.33948.1 (1.57), MSTRG.34175.1 (1.78), MSTRG.35257.14 (6.49), MSTRG.37034.2 (3.80), MSTRG.37910.1 (2.72), MSTRG.38366.1 (3.16), MSTRG.40130.1 (2.53), MSTRG.41437.1 (1.45), MSTRG.4215.1 (1.35), MSTRG.4832.2 (4.35), MSTRG.5140.1 (8.41)
Pav_sc0000910.1_g790.1.mk	1.15	XTH8	MSTRG.33128.1 (1.17), MSTRG.40513.1 (1.42), MSTRG.41450.1 (1.71)
Pav_sc0000308.1_g610.1.mk	1.70	XTH23	MSTRG.25299.1 (3.76), MSTRG.30248.1 (4.10), MSTRG.3112.1 (2.62), MSTRG.33336.1 (1.35), MSTRG.37910.1 (2.72), MSTRG.40130.1 (2.53), MSTRG.4215.1 (1.35)
Pav_sc0000359.1_g040.1.mk	3.67	XTH24	MSTRG.13549.1 (1.91), MSTRG.25299.1 (3.76), MSTRG.30065.2 (−7.24), MSTRG.30248.1 (4.10), MSTRG.3112.1 (2.62), MSTRG.33336.1 (1.35), MSTRG.35294.2 (−4.54), MSTRG.4215.1 (1.35)
Pav_sc0000354.1_g310.1.mk	1.32	XTH33	MSTRG.2169.2 (1.13), MSTRG.30065.2 (−7.24), MSTRG.35294.2 (−4.54), MSTRG.38400.3 (−2.61)
Pav_sc0000582.1_g630.1.mk	2.06	CEL9-like	MSTRG.13879.1 (1.09), MSTRG.30707.15 (−5.26), MSTRG.3112.1 (2.62), MSTRG.33336.1 (1.35), MSTRG.34175.1 (1.78), MSTRG.37034.2 (3.80), MSTRG.37910.1 (2.72), MSTRG.40130.1 (2.53), MSTRG.4215.1 (1.35)
Pav_sc0000652.1_g760.1.mk	1.83	CEL CX	MSTRG.13122.1 (1.26), MSTRG.15876.1 (−1.69), MSTRG.165.3 (1.84), MSTRG.21139.1 (3.20), MSTRG.22209.11 (−4.63), MSTRG.23122.2 (1.24), MSTRG.23545.5 (2.47), MSTRG.29844.2 (1.97), MSTRG.30536.1 (3.65), MSTRG.30707.15 (−5.26), MSTRG.31012.1 (2.79), MSTRG.338.1 (3.11), MSTRG.33948.1 (1.57), MSTRG.37034.2 (3.80), MSTRG.37910.1 (2.72), MSTRG.40130.1 (2.53), MSTRG.41437.1 (1.45), MSTRG.5140.1 (8.41), MSTRG.7786.1 (2.30)
Pav_sc0000244.1_g040.1.mk	1.12	EXP-like A2.1	MSTRG.13549.1 (1.91), MSTRG.22060.23 (2.06), MSTRG.25299.1 (3.76), MSTRG.25638.2 (5.61), MSTRG.30248.1 (4.10), MSTRG.3112.1 (2.62), MSTRG.33336.1 (1.35), MSTRG.37910.1 (2.72), MSTRG.40130.1 (2.53), MSTRG.4215.1 (1.35)
Pav_co4016743.1_g010.1.br	1.32	EXP-like A2.2	MSTRG.22060.23 (2.06), MSTRG.25299.1 (3.76), MSTRG.25638.2 (5.61), MSTRG.30248.1 (4.10), MSTRG.30707.15 (−5.26), MSTRG.3112.1 (2.62), MSTRG.33336.1 (1.35), MSTRG.37034.2 (3.80), MSTRG.37910.1 (2.72), MSTRG.40130.1 (2.53), MSTRG.4215.1 (1.35)
Pav_sc0000582.1_g890.1.mk	−1.76	PG inhibitor 1-like	MSTRG.13879.1 (1.09), MSTRG.18349.5 (−1.23), MSTRG.30248.1 (4.10), MSTRG.30707.15 (−5.26), MSTRG.3112.1 (2.62), MSTRG.33336.1 (1.35), MSTRG.338.1 (3.11), MSTRG.37034.2 (3.80), MSTRG.37910.1 (2.72), MSTRG.40130.1 (2.53), MSTRG.4215.1 (1.35), MSTRG.5140.1 (8.41)
Pav_sc0000843.1_g400.1.mk	−2.52	PME inhibitor2	MSTRG.13122.1 (1.26), MSTRG.15876.1 (−1.69), MSTRG.21139.1 (3.20), MSTRG.22209.11 (−4.63), MSTRG.23122.2 (1.24), MSTRG.29844.2 (1.97), MSTRG.30707.15 (−5.26), MSTRG.31012.1 (2.79), MSTRG.338.1 (3.11), MSTRG.33948.1 (1.57), MSTRG.37034.2 (3.80), MSTRG.37910.1 (2.72), MSTRG.40130.1 (2.53), MSTRG.41437.1 (1.45), MSTRG.5140.1 (8.41), MSTRG.7786.1 (2.30)
Pav_sc0001335.1_g240.1.mk	−1.15	PME inhibitor34	
Pav_sc0000045.1_g280.1.mk	2.14	CHS1-like	MSTRG.13122.1 (1.26), MSTRG.15876.1 (−1.69), MSTRG.21139.1 (3.20), MSTRG.23122.2 (1.24), MSTRG.29844.2 (1.97), MSTRG.30048.2 (3.09), MSTRG.338.1 (3.11), MSTRG.41437.1 (1.45), MSTRG.5140.1 (8.41), MSTRG.7786.1 (2.30)
Pav_sc0007510.1_g020.1.mk	1.33	CHI	MSTRG.13122.1 (1.26), MSTRG.15876.1 (−1.69), MSTRG.165.3 (1.84), MSTRG.21139.1 (3.20), MSTRG.23122.2 (1.24), MSTRG.29844.2 (1.97), MSTRG.31012.1 (2.79), MSTRG.338.1 (3.11), MSTRG.5140.1 (8.41), MSTRG.7786.1 (2.30)
Pav_sc0002208.1_g840.1.mk	1.26	DFR	MSTRG.13122.1 (1.26), MSTRG.15876.1 (−1.69), MSTRG.21139.1 (3.20), MSTRG.23122.2 (1.24), MSTRG.29844.2 (1.97), MSTRG.338.1 (3.11), MSTRG.41437.1 (1.45), MSTRG.5140.1 (8.41), MSTRG.7786.1 (2.30)
Pav_sc0000625.1_g100.1.mk	2.74	MYB44-like	MSTRG.15876.1 (−1.69), MSTRG.1606.1 (1.57), MSTRG.21139.1 (3.20), MSTRG.22209.11 (−4.63), MSTRG.23122.2 (1.24), MSTRG.23545.5 (2.47), MSTRG.29844.2 (1.97), MSTRG.30707.15 (−5.26), MSTRG.338.1 (3.11), MSTRG.33948.1 (1.57), MSTRG.34175.1 (1.78), MSTRG.35257.14 (6.49), MSTRG.37034.2 (3.80), MSTRG.37910.1 (2.72), MSTRG.38366.1 (3.16), MSTRG.40130.1 (2.53), MSTRG.41437.1 (1.45), MSTRG.4215.1 (1.35), MSTRG.4832.2 (4.35), MSTRG.5140.1 (8.41), MSTRG.7786.1 (2.30)
Pav_sc0000766.1_g080.1.mk	1.49	MYB306-like1	MSTRG.2169.2 (1.13), MSTRG.30065.2 (−7.24), MSTRG.33128.1 (1.17), MSTRG.35294.2 (−4.54), MSTRG.38400.3 (−2.61), MSTRG.40513.1 (1.42), MSTRG.41450.1 (1.71), MSTRG.41822.2 (1.17)
Pav_sc0000877.1_g610.1.mk	1.59	MYB306-like2	MSTRG.23059.1 (−3.96), MSTRG.38366.1 (3.16)
Pav_sc0000464.1_g210.1.br	1.11	MYB10 V1-3	MSTRG.13122.1 (1.26), MSTRG.15876.1 (−1.69), MSTRG.29844.2 (1.97), MSTRG.31012.1 (2.79), MSTRG.338.1 (3.11), MSTRG.5140.1 (8.41), MSTRG.7786.1 (2.30),
Pav_sc0000586.1_g190.1.mk	1.87	bHLH13	MSTRG.23059.1 (−3.96)
Pav_sc0000624.1_g1350.1.mk	2.66	bHLH35	MSTRG.2169.2 (1.13), MSTRG.30065.2 (−7.24), MSTRG.33128.1 (1.17), MSTRG.35294.2 (−4.54), MSTRG.38400.3 (−2.61)
Pav_sc0000143.1_g320.1.mk	−1.48	bHLH51	MSTRG.13122.1 (1.26), MSTRG.15876.1 (−1.69), MSTRG.21139.1 (3.20), MSTRG.30707.15 (−5.26), MSTRG.338.1 (3.11), MSTRG.41437.1 (1.45), MSTRG.4882.1 (−1.47), MSTRG.5140.1 (8.41), MSTRG.7786.1 (2.30)
Pav_sc0001519.1_g020.1.mk	1.29	bHLH74	MSTRG.13879.1 (1.09), MSTRG.23545.5 (2.47), MSTRG.3112.1 (2.62), MSTRG.33336.1 (1.35), MSTRG.37034.2 (3.80), MSTRG.37910.1 (2.72), MSTRG.4215.1 (1.35)
Pav_sc0000998.1_g640.1.mk	3.00	bHLH92	MSTRG.40513.1 (1.42)
Pav_sc0001422.1_g020.1.mk	1.02	bHLH113	MSTRG.15876.1 (−1.69), MSTRG.1606.1 (1.57), MSTRG.21139.1 (3.20), MSTRG.30707.15 (−5.26), MSTRG.3112.1 (2.62), MSTRG.338.1 (3.11), MSTRG.35257.14 (6.49), MSTRG.37034.2 (3.80), MSTRG.37910.1 (2.72), MSTRG.38366.1 (3.16), MSTRG.40130.1 (2.53), MSTRG.4215.1 (1.35), MSTRG.5140.1 (8.41)
Pav_sc0001313.1_g380.1.mk	3.07	bHLH162-like1	MSTRG.30065.2 (−7.24), MSTRG.35294.2 (−4.54), MSTRG.38400.3 (−2.61), MSTRG.9626.1 (3.95)
Pav_sc0001309.1_g720.1.mk	3.41	bHLH162-like2	MSTRG.13549.1 (1.91), MSTRG.25299.1 (3.76), MSTRG.27214.15 (−2.50), MSTRG.30065.2 (−7.24), MSTRG.3112.1 (2.62), MSTRG.35294.2 (−4.54)
Pav_sc0006499.1_g050.1.mk	2.15	MYC2	MSTRG.13879.1 (1.09), MSTRG.1606.1 (1.57), MSTRG.21139.1 (3.20), MSTRG.23122.2 (1.24), MSTRG.23545.5 (2.47), MSTRG.29844.2 (1.97), MSTRG.30707.15 (−5.26), MSTRG.3112.1 (2.62), MSTRG.338.1 (3.11), MSTRG.33948.1 (1.57), MSTRG.34175.1 (1.78), MSTRG.37034.2 (3.80), MSTRG.37910.1 (2.72), MSTRG.38366.1 (3.16), MSTRG.40130.1 (2.53), MSTRG.4215.1 (1.35), MSTRG.4832.2 (4.35), MSTRG.5140.1 (8.41)
Pav_sc0001557.1_g070.1.mk	3.08	WD40.1	MSTRG.25299.1 (3.76), MSTRG.30065.2 (−7.24), MSTRG.3112.1 (2.62), MSTRG.35294.2 (−4.54), MSTRG.38400.3 (−2.61)
Pav_sc0001557.1_g120.1.br	3.05	WD40.2	
Pav_sc0001963.1_g370.1.mk	1.42	WD40.3	MSTRG.30065.2 (−7.24), MSTRG.35294.2 (−4.54), MSTRG.38400.3 (−2.61)

The number following DE-lncRNA represents the value of log_2_ (fold change of this lncRNA).

**Table 4:** Pearson’s correlation analysis of the major anthocyanin abundance and the expression level of related DEGs/DE-lncRNAs.

	Cyanidin 3-Rutinoside	Cyanin Chloride	Cyanidin 3-O-Galactoside	Peonidin 3-O-Glucoside Chloride	Cyanidin 3-O-Glucoside	Cyanidin O-Syringic Acid
*CYP707A1-like*	0.667 *	0.788 *	0.582	0.726 *	0.591 *	0.506
*CYP707A4-like1*	−0.813 *	−0.691 *	−0.858 *	−0.630 *	−0.850 *	−0.882 *
*CYP707A4-like2*	−0.849 *	−0.742 *	−0.893 *	−0.692 *	−0.887 *	−0.915 *
*CHS1-like*	0.894 *	0.965 *	0.848 *	0.968 *	0.874 *	0.839 *
*CHI*	0.895 *	0.913 *	0.836 *	0.847 *	0.845 *	0.813 *
*CHI3*	0.981 *	0.956 *	0.973 *	0.902 *	0.986 *	0.977 *
*DFR*	0.964 *	0.980 *	0.930 *	0.937 *	0.945 *	0.921 *
*MYB10.1*	0.971 *	0.953 *	0.967 *	0.932 *	0.980 *	0.979 *
*MYB44-like*	0.818 *	0.910 *	0.751 *	0.860 *	0.762 *	0.695 *
*MYB306-like1*	−0.292	−0.105	−0.392	−0.077	−0.374	−0.451
*MYB306-like2*	0.498	0.577	0.420	0.487	0.401	0.321
*MYB10 V1-3*	0.967 *	0.962 *	0.927 *	0.875 *	0.937 *	0.907 *
*bHLH13*	0.118	0.255	0.031	0.252	0.050	−0.020
*bHLH33*	−0.890 *	−0.799 *	−0.921 *	−0.754 *	−0.925 *	−0.952 *
*bHLH35*	−0.238	−0.058	−0.333	−0.066	−0.327	−0.418
*bHLH51*	−0.833 *	−0.903 *	−0.759 *	−0.848 *	−0.787 *	−0.731 *
*bHLH74*	0.540	0.684 *	0.435	0.629 *	0.465	0.376
*bHLH92*	−0.788 *	−0.657 *	−0.849 *	−0.613 *	−0.842 *	−0.886 *
*bHLH113*	0.696 *	0.829 *	0.622 *	0.801 *	0.648 *	0.571
*bHLH162-like1*	−0.001	0.179	−0.115	0.160	−0.103	−0.197
*bHLH162-like2*	0.200	0.392	0.096	0.412	0.124	0.039
*MYC2*	0.696 *	0.810 *	0.609 *	0.756 *	0.620 *	0.542
*WD40.1*	0.180	0.365	0.064	0.366	0.091	0.004
*WD40.2*	0.242	0.360	0.131	0.251	0.131	0.036
*WD40.3*	−0.083	0.098	−0.197	0.088	−0.178	−0.267
*MSTRG.11213.10*	0.780 *	0.650 *	0.831 *	0.606 *	0.829 *	0.874 *
*MSTRG.13193.1*	0.687 *	0.547	0.738 *	0.496	0.744 *	0.797 *
*MSTRG.15876.1*	−0.856 *	−0.930 *	−0.798 *	−0.899 *	−0.815 *	−0.765 *
*MSTRG.1606.1*	0.637 *	0.757 *	0.563	0.714 *	0.582 *	0.505
*MSTRG.165.3*	0.777 *	0.815 *	0.704 *	0.762 *	0.711 *	0.674 *
*MSTRG.21139.1*	0.813 *	0.899 *	0.738 *	0.865 *	0.761 *	0.707 *
*MSTRG.22209.11*	−0.837 *	−0.890 *	−0.788 *	−0.846 *	−0.795 *	−0.751 *
*MSTRG.23122.2*	0.842 *	0.893 *	0.785 *	0.841 *	0.788 *	0.741 *
*MSTRG.23545.5*	0.709 *	0.782 *	0.609 *	0.675 *	0.620 *	**0.544**
*MSTRG.25299.1*	0.313	0.490	0.224	0.527	0.260	0.188
*MSTRG.26521.2*	−0.859 *	−0.769 *	−0.897 *	−0.742 *	−0.905 *	−0.937 *
*MSTRG.27214.15*	−0.233	−0.357	−0.134	−0.296	−0.180	−0.096
*MSTRG.29836.1*	0.949 *	0.900 *	0.921 *	0.791 *	0.927 *	0.911 *
*MSTRG.29844.2*	0.829 *	0.886 *	0.747 *	0.791 *	0.761 *	0.699 *
*MSTRG.30048.2*	0.716 *	0.845 *	0.689 *	0.929 *	0.728 *	0.693 *
*MSTRG.30065.2*	0.010	−0.182	0.129	−0.174	0.102	0.196
*MSTRG.3040.1*	−0.809 *	−0.678 *	−0.857 *	−0.581	−0.844 *	−0.867 *
*MSTRG.30707.15*	−0.723 *	−0.820 *	−0.644 *	−0.769 *	−0.661 *	−0.596 *
*MSTRG.30960.1*	−0.828 *	−0.712 *	−0.877 *	−0.672 *	−0.874 *	−0.913 *
*MSTRG.31012.1*	0.839 *	0.860 *	0.758 *	0.717 *	0.755 *	0.690 *
*MSTRG.3112.1*	0.454	0.636 *	0.363	0.671 *	0.397	0.320
*MSTRG.33128.1*	−0.503	−0.323	−0.587 *	−0.259	−0.570	−0.630 *
*MSTRG.33336.1*	0.502	0.651 *	0.388	0.600 *	0.425	0.334
*MSTRG.33370.3*	−0.891 *	−0.814 *	−0.924 *	−0.732 *	−0.924 *	−0.926 *
*MSTRG.338.1*	0.867 *	0.946 *	0.805 *	0.906 *	0.821 *	0.765 *
*MSTRG.33948.1*	0.856 *	0.902 *	0.790 *	0.787 *	0.793 *	0.724 *
*MSTRG.34175.1*	0.658 *	0.731 *	0.578	0.597 *	0.562	0.470
*MSTRG.34331.1*	−0.841 *	−0.738 *	−0.881 *	−0.690 *	−0.874 *	−0.901 *
*MSTRG.35257.14*	0.714 *	0.825 *	0.682 *	0.825 *	0.694 *	0.631 *
*MSTRG.35294.2*	0.009	−0.178	0.118	−0.185	0.101	0.187
*MSTRG.36057.7*	−0.707 *	−0.569	−0.769 *	−0.537	−0.767 *	−0.821 *
*MSTRG.36335.1*	−0.760 *	−0.663 *	−0.797 *	−0.646 *	−0.791 *	−0.828 *
*MSTRG.37034.2*	0.729 *	0.820 *	0.633 *	0.741 *	0.646 *	0.574
*MSTRG.37910.1*	0.613 *	0.758 *	0.526	0.758 *	0.555	0.487
*MSTRG.38366.1*	0.702 *	0.782 *	0.624 *	0.668 *	0.621 *	0.534
*MSTRG.38400.3*	0.045	−0.133	0.166	−0.115	0.151	0.243
*MSTRG.40130.1*	0.721 *	0.829 *	0.638 *	0.771 *	0.649 *	0.573
*MSTRG.40513.1*	−0.563	−0.384	−0.646 *	−0.314	−0.626 *	−0.683 *
*MSTRG.41437.1*	0.822 *	0.909 *	0.761 *	0.895 *	0.786 *	0.736 *
*MSTRG.41450.1*	−0.462	−0.308	−0.539	−0.285	−0.531	−0.593 *
*MSTRG.41822.2*	−0.198	0.009	−0.301	0.106	−0.254	−0.312
*MSTRG.4215.1*	0.587 *	0.734 *	0.485	0.701 *	0.514	0.429
*MSTRG.4832.2*	0.776 *	0.828 *	0.705 *	0.691 *	0.695 *	0.613 *
*MSTRG.4882.1*	−0.801 *	−0.863 *	−0.740 *	−0.808 *	−0.767 *	−0.716 *
*MSTRG.5140.1*	0.855 *	0.936 *	0.786 *	0.892 *	0.804 *	0.747 *
*MSTRG.7786.1*	0.872 *	0.930 *	0.804 *	0.889 *	0.831 *	0.789 *
*MSTRG.8543.2*	−0.898 *	−0.817 *	−0.921 *	−0.777 *	−0.930 *	−0.956 *
*MSTRG.9626.1*	0.090	0.241	−0.032	0.192	−0.021	−0.110
*MSTRG.10166.2*	0.826 *	0.713 *	0.862 *	0.661 *	0.863 *	0.899 *
*MSTRG.12848.1*	0.959 *	0.934 *	0.932 *	0.806 *	0.930 *	0.893 *
*MSTRG.13122.1*	0.819 *	0.884 *	0.744 *	0.842 *	0.771 *	0.726 *
*MSTRG.13549.1*	0.343	0.505	0.269	0.566	0.301	0.246
*MSTRG.13879.1*	0.476	0.602 *	0.350	0.499	0.376	0.279
*MSTRG.2169.2*	−0.392	−0.202	−0.463	−0.140	−0.451	−0.522
*MSTRG.23059.1*	−0.431	−0.532	−0.360	−0.521	−0.368	−0.309

The number in the table represents the correlation coefficient. Positive number indicates positive correlation. Negative number indicates negative correlation. Asterisk indicates a significant level of correlation (* *p* < 0.05).

## Data Availability

All data are provided in the article and the Supplementary Materials.

## Supplementary Materials

The following supporting information can be downloaded at: https://www.mdpi.com/article/10.3390/ijms25189860/s1.
